# Study of Spatial and Temporal Variations of Ionospheric Total Electron Content in Japan, during 2014–2019 and the 2016 Kumamoto Earthquake

**DOI:** 10.3390/s21062156

**Published:** 2021-03-19

**Authors:** Tianyang Hu, Yibin Yao, Jian Kong

**Affiliations:** 1 School of Geodesy and Geomatics, Wuhan University, Wuhan 430079, China; hutianyang@whu.edu.cn; 2 Chinese Antarctic Center of Surveying and Mapping, Wuhan University, Wuhan 430079, China; jkong@whu.edu.cn

**Keywords:** total electron content (TEC), spherical cap harmonic (SCH), spatial and temporal variations, ionospheric disturbances, 2016 Kumamoto earthquake, ionization blobs

## Abstract

There are a large number of excellent research cases in Global Navigation Satellite System (GNSS) positioning and disaster prediction in Japan region, where the simulation and prediction of total electron content (TEC) is a powerful research method. In this study, we used the data of the GNSS Earth Observation Network (GEONET) established by the Geographical Survey Institute of Japan (GSI) to compare the performance of two regional ionospheric models in Japan, in which the spherical cap harmonic (SCH) model has the best performance. In this paper, we investigated the spatial and temporal variations of ionospheric TEC in Japan and their relationship with latitude, longitude, seasons, and solar activity. The results show that the TEC in Japan increases as the latitude decreases, with the highest average TEC in spring and summer and the lowest in winter, and has a strong correlation with solar activity. In addition, the observation and analysis of ionospheric disturbances over Japan before the 2016 Kumamoto earthquake and geomagnetic storms showed that GNSS observing of ionospheric TEC seems to be very effective in forecasting natural disasters and monitoring space weather.

## 1. Introduction

Ionospheric delay can be one of the main sources of error in Global Navigation Satellite System (GNSS) navigation and positioning, and its impact can reach up to 100 m. The number of free electrons in the ionosphere is measured by total electron content (TEC), which is one of the most important ionospheric parameters. Ionospheric TEC is proportional to the GNSS ionospheric delay [1,2] and changes continuously with space, time, solar, and geomagnetic activities. TEC can be calculated from GNSS dual-frequency observations and used to establish global or regional ionospheric models [2,3,4,5] to improve the performance of GNSS positioning. A great number of institutions and scholars have used various forecasting models and mapping algorithms to study ionospheric variations. Global ionospheric models include the Global Ionospheric Map (GIM) provided by International GNSS Service (IGS) and Centre for Orbit Determination in Europe (CODE), the International Reference Ionospheric Model (IRI), and Klobuchar, NeQuick, etc. Regional ionospheric models include polynomial model (POLY), spherical cap harmonic function (SCH), auto-regressive moving average (ARMA), etc. [6,7,8]. However, different models have different performance and spatial resolutions, and their applications in different regions need to be verified by further work.

Many scholars have evaluated the accuracy of the ionospheric TEC models. Hernández-Pajares et al. [9] analyzed the electron content distribution of the north and south polar ionosphere from 2001 to the beginning of 2019 by using the global ionospheric map (GIM) of VTEC (vertical TEC) computed from GNSS by UPC-IonSAT with a tomographic-kriging combined technique (UQRG GIM), and achieved better results than other methods. Ansari K et al. [10] improved the linear time-series model (LTM) and achieved better accuracy than ARMA in southwest Japan. Venkata Ratnam D et al. [11] used a model based on spherical harmonic function (SHF) for modeling the ionospheric TEC in low-latitude regions like as India, and the results indicate that the SHF model is capable of estimating the ionospheric delays well. This shows that these models perform better under appropriate conditions. K. Nishimoto [12] applied the spherical cap harmonic function analysis (SCHA) to investigate the VTEC (vertical TEC) distribution in Japan in 2011 and discussed its capability for ionospheric model prediction. In this study, we tried to compare the suitability of the polynomial (POLY) model and spherical cap harmonic function (SCH) model in Japan adopting ground-based GNSS measurements as a supplement to the current research on the regional ionospheric models.

Japan is located in the mid- and low latitudes, where ionospheric activities are frequent. They are not only closely related to space and time, but also extremely easily affected by natural events such as earthquakes and geomagnetic storms. These events are widely observed and provide good research cases for studying various ionospheric variations [13]. Earthquakes and other natural disasters occur frequently in Japan, and there are often different types of widely observed and analyzed ionospheric disturbances that last for a period of time before the earthquake [14,15,16], which may be used as important bases for analyzing and forecasting earthquakes. When a geomagnetic storm occurs, strong ionospheric density irregularities are generated, showing depletions (bubbles), enhancements (blobs), and scintillation [17,18], and they are all formed in the F layer of the ionosphere [19]. It is of great significance to study the formation and evolution of ionospheric disturbances [20]. At present, GNSS is widely used to observe ionospheric disturbances caused by earthquakes and space weather events, and can be used to monitor such phenomena by analyzing variations of ionospheric TEC. Another goal of this study is to use the GNSS Earth Observation Network (GEONET) data to map the TEC disturbances in Japan, providing a case for the use of ionosphere for disaster warning and some useful conclusions.

In this paper, we used a polynomial model and spherical cap harmonic function model to simulate TEC in Japan and compared their accuracy. Then, we studied the spatial and temporal variation characteristics of ionospheric TEC in Japan and its relationship with solar and geomagnetic activities. Our analysis of the ionospheric TEC disturbances before the 2016 Kumamoto earthquake is outlined in Section 5, and the observation and detection results of the plasma blobs in Japan on 8 September 2017 are discussed in Section 6.

## 2. Materials and Methods

### 2.1. GEONET Data

**Figure 1:**
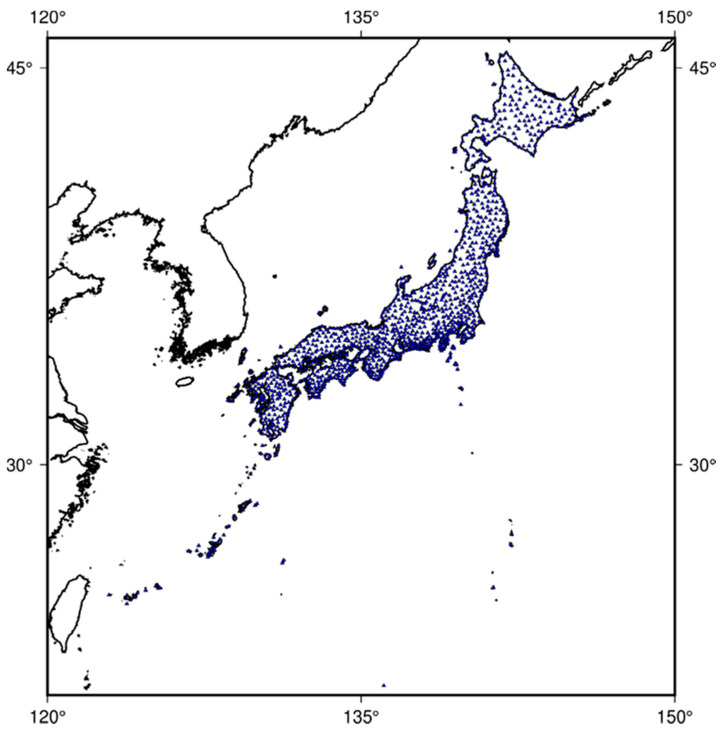
Distribution of GNSS Earth Observation Network (GEONET) composed of more than 1300 GPS permanent receivers (as of November 2020).

GNSS Earth Observation Network (GEONET), the Japanese nationwide GNSS receiver network, has been operated by the Geographical Survey Institute of Japan since 1994 [21]. It has expanded into a dense network of more than 1300 permanent receivers by November 2020, one of the densest GNSS receiver networks on earth. Its distribution is shown in the Figure 1. The two-dimensional ionospheric disturbances over Japan can be mapped with GEONET high-resolution observation data [22]. These receivers are located at an average interval of about 25 km and continuously provide GNSS data at a sampling rate of 30 s [23]. GEONET has been used for long-term observation and monitoring of crustal deformation [10,24], and has been used to solve global and regional issues such as earthquake forecasting, disaster management, high-precision crustal deformation, and strain analysis. It can also be used to evaluate performance and suitability of a regional ionospheric model. The 30 s sampling rate data are available via ftp://terras.gsi.go.jp (accessed on 15 March 2021).

### 2.2. Calculating TEC by GEONET GNSS Measurements

GNSS observations include two types: pseudo ranging and carrier phase surveying. The basic formulae are as follows:

(1) $$P_{k}^{i,j}=\rho +c\left(\delta t_{T}-\delta t^{R}\right)+c\left(\delta r_{T}-\delta r^{R}\right)+I_{k}^{T,R}+Tr_{k}^{T,R}+B_{q,k}^{T}+B_{q,k}^{R}+M_{k}^{T,R}+B_{a}+\varepsilon_{k}$$

(2) $$\lambda_{k}\phi_{k}^{T,R}\left(t\right)=\rho +\lambda_{k}N_{k}+c\left(\delta t_{T}-\delta t^{R}\right)+c\left(\delta r_{T}-\delta r^{R}\right)-I_{k}^{T,R}+Tr_{k}^{T,R}+b_{q,k}^{T}+b_{q,k}^{R}+m_{k}^{T,R}+e_{k}$$

where *P* is the pseudo range observations; *T* and *R* denote the satellites and receivers; *k* = 1,2 are the carriers L1 and L2; *ρ* is the geometric distance between the satellite and receiver antenna; $\delta t_{T}$ and $\delta t^{R}$ are the clock error of the satellite and receiver; $\delta r_{T}$ and $\delta r^{R}$ are the relativistic effect of the satellite and receiver; *c* is the speed of light in vacuum; *I* is the ionospheric refractive error; *Tr* is the tropospheric refractive error; $B_{q}$ is the group delay of the satellite and receiver; $M_{k}^{T,R}$ is the multipath effects; $B_{a}$ is the displaced phase centers antenna (DPCA); *ε* is the noise of pseudo-range measurement.

Ignoring *i* and *j*, we calculate the difference between frequencies in Formula (1). It can be expressed as follows:

(3) $$P_{1}-P_{2}=\left(1-\gamma \right)I_{1}+B^{T}+B^{R}+\left(M_{1}-M_{2}\right)+\varepsilon_{1,2}$$

where $\varepsilon_{1,2}$ is the difference between two noises of pseudo-range measurement between different frequencies; $B^{T}=B_{q,1}^{T}-B_{q,2}^{T}$ and $B^{R}=B_{q,1}^{R}-B_{q,2}^{R}$ are the satellite and receiver different code biases (DCBs).

When there is a proper elevation mask, the multipath effects can be ignored. The ionospheric delay at L1 frequency can be expressed as:

(4) $$I_{1}=\frac{40.3}{f_{1}^{2}}TEC$$

and we can substitute Formula (4) into Formula (3) to get the expression of TEC:

(5) $$TEC_{P}=\frac{f_{1}^{2}f_{2}^{2}}{40.3\left(f_{2}^{2}-f_{1}^{2}\right)}\left(P_{1}-P_{2}-B^{T}-B^{R}\right)$$

where *p* is the pseudo range observations; $P_{1}-P_{2}$ is the dual-frequency observations. The DCBs are going to be estimated by the fitting models in Section 2.3.

However, there are different *TEC* from the same station to different satellites, which is recorded as *STEC* (slant *TEC*). *STEC* is related to the satellite elevation angle and the smaller the angle, the larger the *STEC*. To be more rigorous and accurate, the *TEC* in vertical direction is often used in research and is denoted as *VTEC* (vertical *TEC*), which is out of relationship with satellite elevation angle. *STEC* can be converted to *VTEC* by the mapping function, which can be expressed as follows:

(6) $$VTEC=STEC\times \cos z^{\prime }=STEC\times \sqrt{1-\sin^{2}z^{\prime }}$$

(7) $$\sin z^{\prime }=\frac{R}{R+H}\sin z$$

where *R* is the mean earth radius; *H* is the single ionospheric height; *z′* is the satellite zenith angle at the ionosphere pierce point (IPP); *z* is the satellite zenith angle at the receiver. In this paper, we only use *VTEC* in the follow analysis.

### 2.3. Method of Modeling

The mapping and forecasting of ionospheric TEC with stable and high-accuracy methods has always been a positive challenge. The global ionospheric map data released by IGS has a spatial resolution of 2.5° × 5° and a temporal resolution of 2 h. It is considered to be the most accurate ionospheric product based on spherical harmonic function in the world [25] and has been a very reliable source of ionospheric TEC information since 1998 [26,27]. Ansari K et al. [28] analyzed the accuracy of the GIM model in Japan. However, we can obtain higher accuracy than GIM and empirical ionospheric models by establishing a regional model in Japan, and can draw images with high spatiotemporal resolution. There are polynomial (POLY) models and spherical cap harmonic function (SCH) models commonly used in regional TEC mapping.

The polynomial model is a relatively simple linear model, and TEC is regarded as a linear combination of a series of factors such as latitude and sun angle. Its expression is as follows:

(8) $$VTEC=\sum_{i=0}^{n}\sum_{k=0}^{m}E_{ik}{\left(\varphi -\varphi^{0}\right)}^{i}{\left(S-S_{0}\right)}^{k}$$

where *VTEC* is the vertical TEC (*VTEC*) at IPP; $E_{ik}$ is the coefficient of model; *n* and *m* are the polynomial orders; $S_{0}$ is the solar time angle of the center point $\left(\varphi_{0},\lambda_{0}\right)$ of the survey area at time $t_{0}$; $S-S_{0}=\left(\lambda -\lambda_{0}\right)+\left(t-t_{0}\right)$; *S* is the solar time angle of the IPP; λ is the geographic latitude of the intersection of the signal path and the single layer; *t* is the observation time.

The spherical cap harmonic function can precisely model the global ionospheric TEC [1,2,29]. Spherical cap harmonics construct a set of orthogonal bases, and any function on the spherical surface can be expressed as a linear combination of spherical cap harmonic functions. Its expression is as follows:

(9) $$VTEC\left(\varphi ,s\right)=\sum_{n=0}^{n_{\max}}\sum_{m=0}^{n}P_{nm}\left(\sin\varphi \right)\left(a_{nm}\cos\left(ms\right)+b_{nm}\sin\left(ms\right)\right)$$

where φ is the geomagnetic latitude of the IPP; $s=\lambda -\lambda_{0}$ denotes the sun-fixed longitude of the IPP; λ signifies the geomagnetic longitude of the IPP, $\lambda_{0}$ signifies the geomagnetic longitude of the sun; *n_max_* is the maximum degree of the model; $P_{nm}$ is a fully normalized associated Legendre function of degree *n* and order *m*; $a_{nm}$ and $b_{nm}$ are the SCH coefficients.

The models mentioned above have different suitability and performance in different regions. Nishimoto K et al. [30] evaluated the suitability of the spherical cap harmonic function model in Japan. However, there is no analysis of the accuracy of these models in Japan. In this paper, the ionospheric thin shell height and the elevation mask are set to 400 km and 15°, respectively. In Section 3, we use the polynomial and the spherical cap harmonic function to model the ionospheric TEC in Japan and compare their performance with the interpolation of GIM.

## 3. Comparison of the Accuracy of the Models

**Figure 2:**
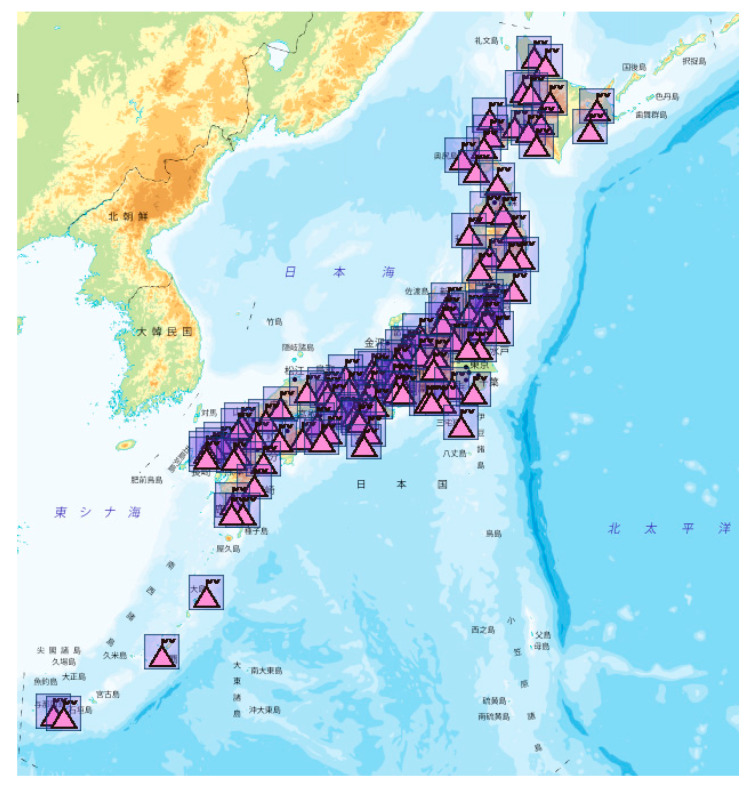
Locations of 100 verification stations in Japan.

In this section, we compared the performance of the two models in Japan. The 10 days GEONET dual-frequency observation data from 9 to 18 June 2019 were selected for modeling, and 100 receivers located throughout Japan were selected as verification stations. The locations of these sites are shown in Figure 2.

In this paper, the bias and root-mean-square (RMS) error between the results of test stations and verification stations are utilized to evaluate the accuracy of these models. They were calculated as the following approximations:

(10) $$bias=\frac{1}{N}\sum_{i=1}^{N}\left(V_{i}-\hat{V_{i}}\right)$$

(11) $$RMS=\sqrt{\frac{\sum_{i=1}^{N}{\left(V_{i}-\hat{V_{i}}\right)}^{2}}{N}}$$

where $V_{i}$ is the result of testing stations; $\hat{V_{i}}$ is the result of verification stations.

**Figure 3:**
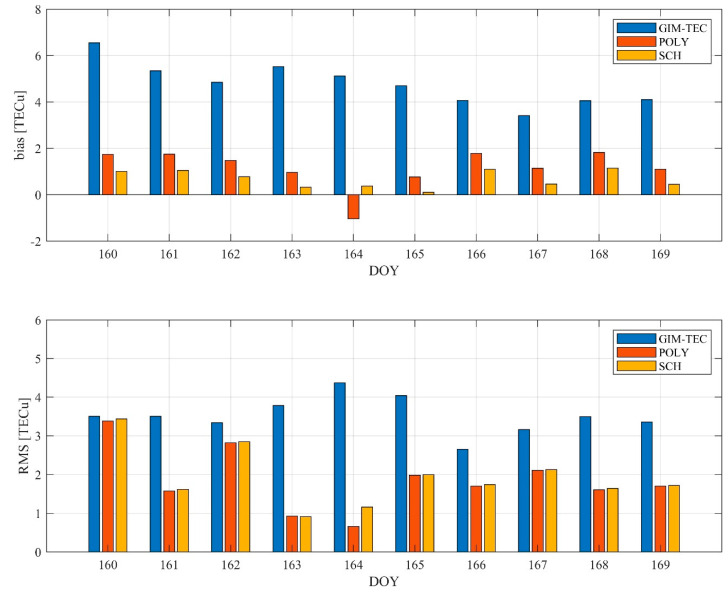
Ionospheric TEC average bias and RMS of the POLY model and the SCH model in Japan from DOY 160 to DOY 169 in 2019.

Figure 3 shows the average bias and RMS error of the two models in Japan, respectively. It can be seen from the figure that the average bias and *RMS* errors of the polynomial model and the spherical cap harmonic function model are within 2.0 TECu and 1.2 TECu, respectively, which are significantly better than GIM. The accuracy of the GIM model is slightly higher at mid-latitude than at low latitude [31]. Here the SCH model performs best in Japan, with the RMS errors less than 3.5 TECu.

**Figure 4:**
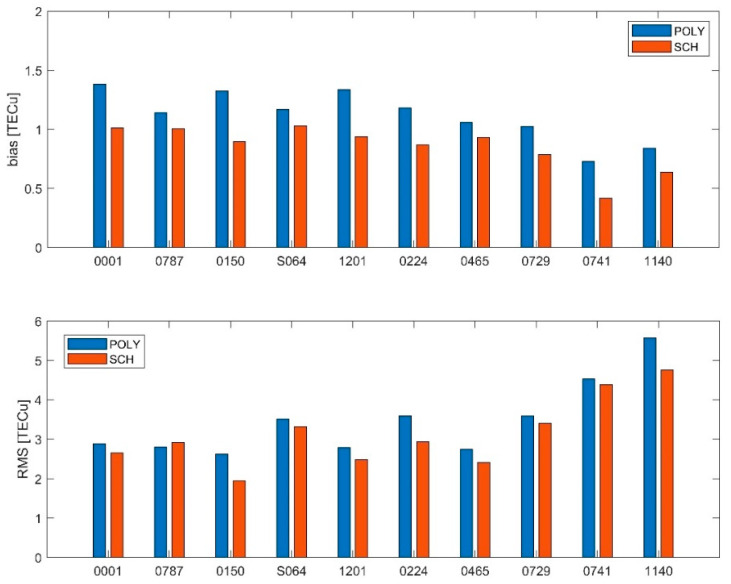
Stational errors of the POLY model and the SCH model in Japan from DOY 160 to DOY 169 in 2019.

The average bias and RMS errors of 10 stations evenly distributed in Japan according to latitude were calculated in order to investigate the variation of the accuracy of the model with latitude. The results are shown in Figure 4. The stations are arranged from high latitude to low latitude. The average bias of the polynomial model and the spherical cap harmonic function model are in the range of 0.5–1 TECu. The RMS errors of the mid-latitude stations are lower than the RMS errors of the low-latitude stations, while it tends to increase as the latitude decreases.

## 4. Spatial and Temporal Variation Characteristics of TEC in Japan Regions

### 4.1. Spatial Variation Characteristics of TEC in Japan Regions

We selected the TECs of DOY080 (spring equinox), DOY172 (summer solstice), DOY266 (autumn equinox), and DOY356 (winter solstice) in 2014 and 2019 in the 24th solar cycle to investigate the spatial variation characteristics of Japanese TEC affected by different factors, including day and night, latitude and longitude, and solar activity. The data of DOY080 in 2014 is missing due to the lack of enough satellites to calculate the ionospheric TEC on that day. The solar activity index of F10.7 on DOY172, DOY266, and DOY356 in 2014 were 101.2, 138.2, and 179.2 sfu, respectively, which is a typical high solar activity year. The F10.7 index on DOY080, DOY172, DOY266, and DOY356 in 2019 were 80.0, 66.4, 66.1, and 71.0 sfu, respectively, which is a typical low solar activity year.

**Figure 5:**
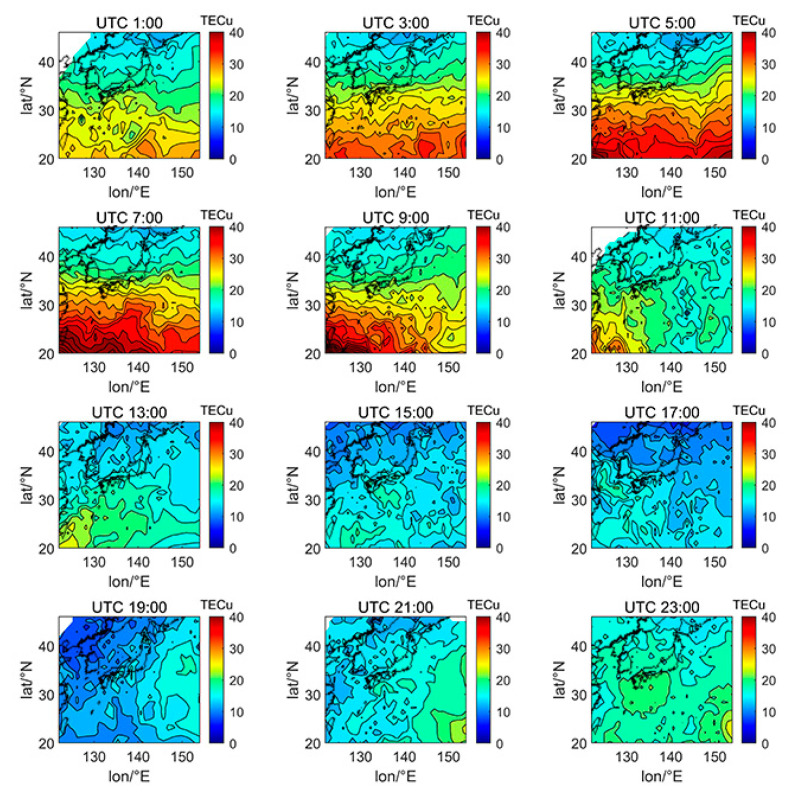
Spatial distribution of TEC in Japan at 2-h intervals in DOY172 in 2014.

**Figure 6:**
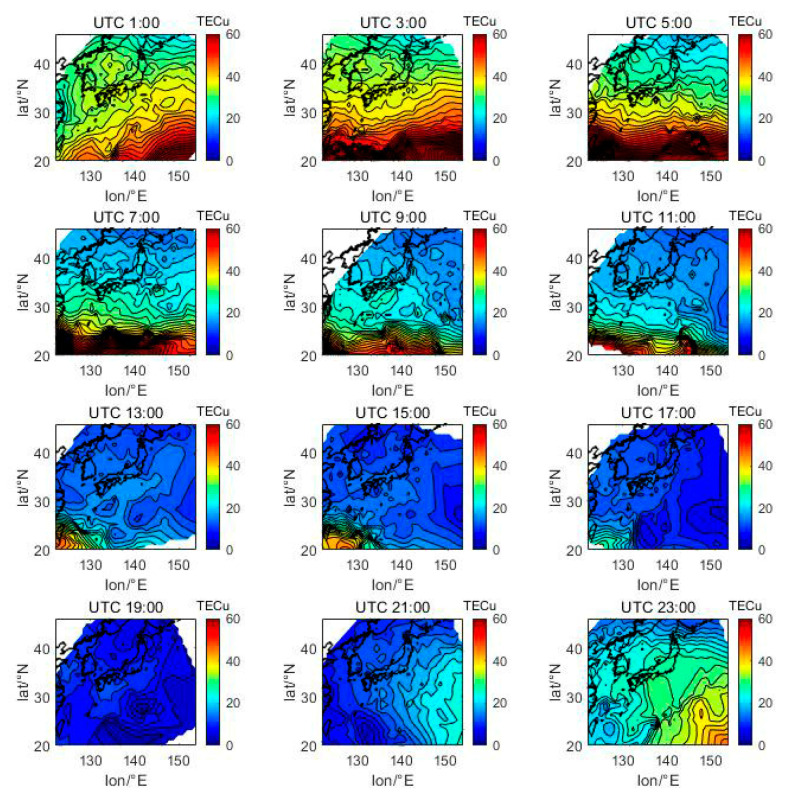
Spatial distribution of TEC in Japan at 2-h intervals in DOY266 in 2014.

**Figure 7:**
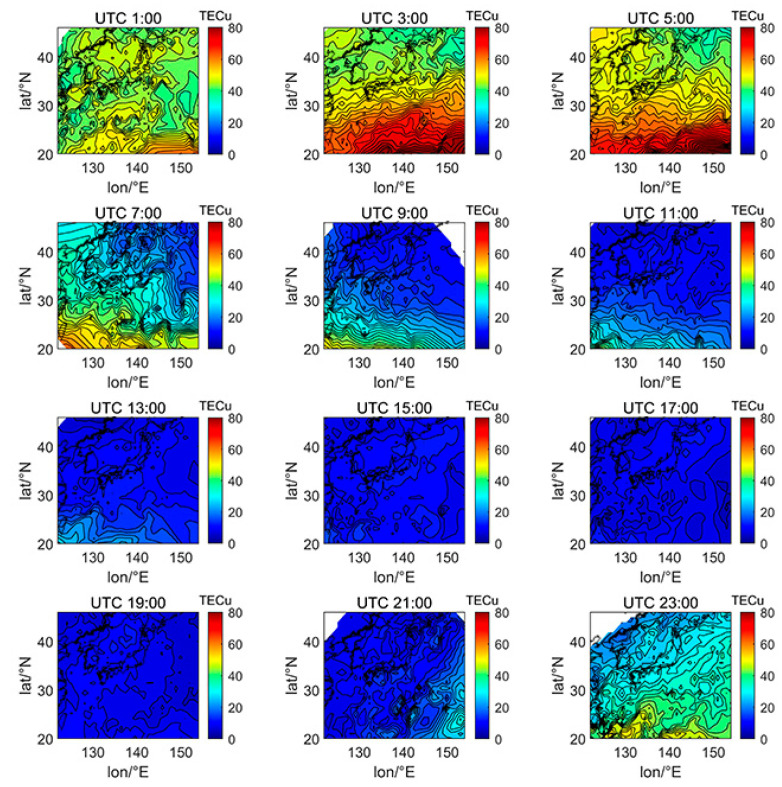
Spatial distribution of TEC in Japan at 2-h intervals in DOY356 in 2014.

**Figure 8:**
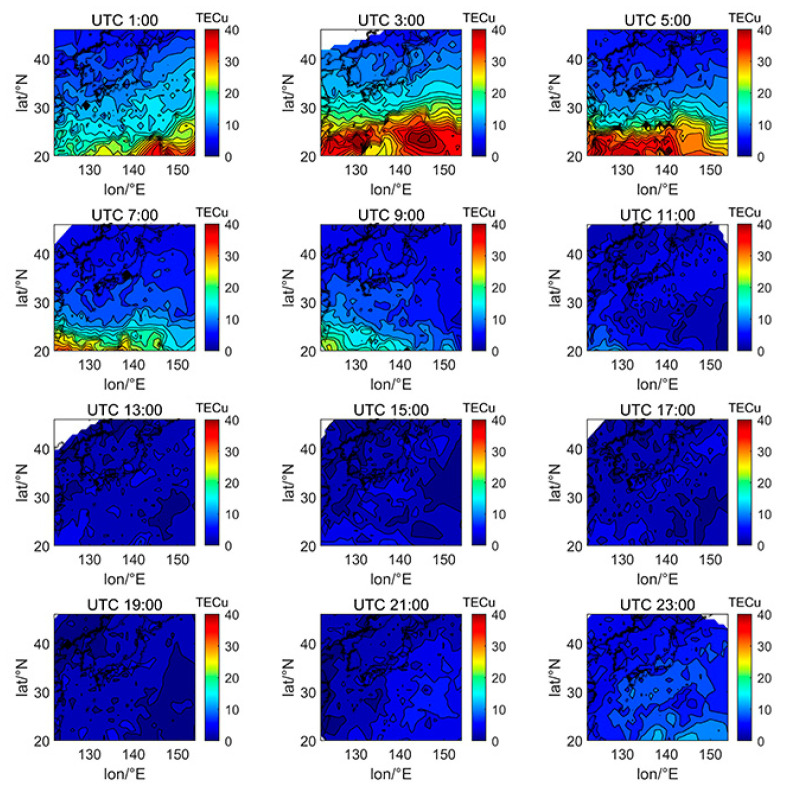
Spatial distribution of TEC in Japan at 2-h intervals in DOY080 in 2019.

**Figure 9:**
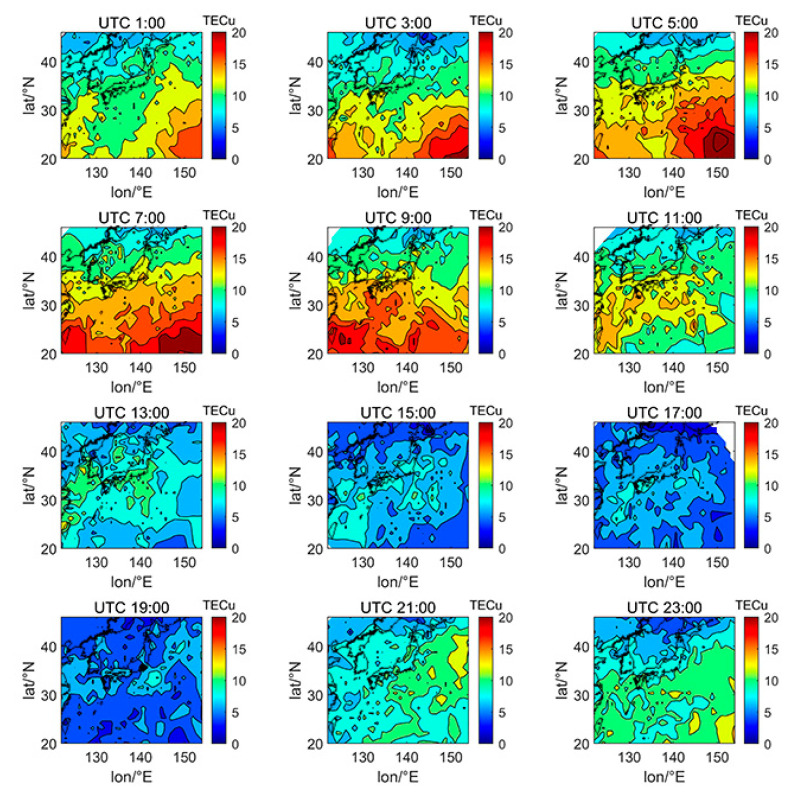
Spatial distribution of TEC in Japan at 2-h intervals in DOY172 in 2019.

**Figure 10:**
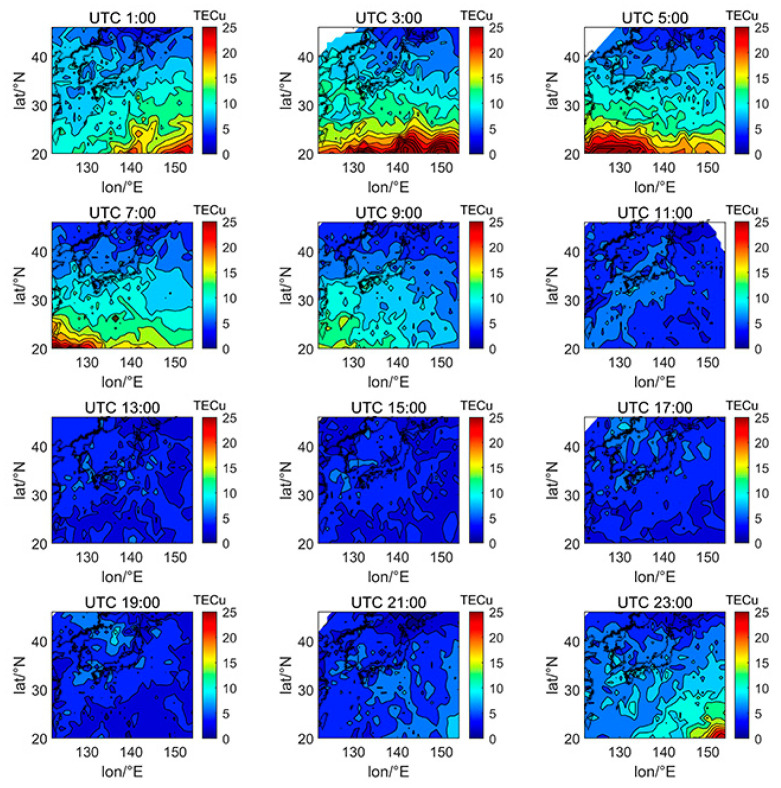
Spatial distribution of TEC in Japan at 2-h intervals in DOY266 in 2019.

**Figure 11:**
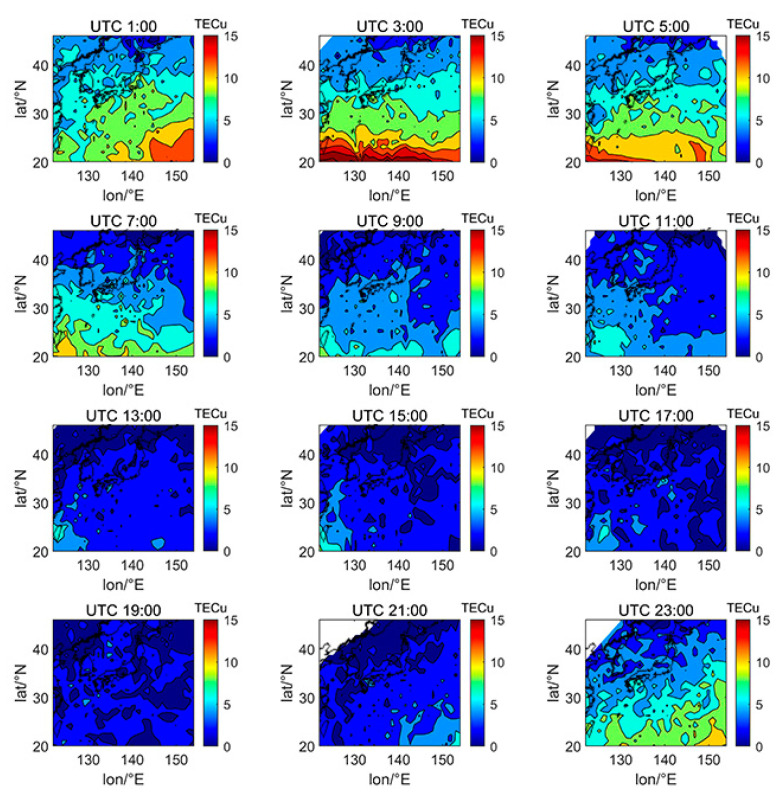
Spatial distribution of TEC in Japan at 2-h intervals in DOY356 in 2019.

Figure 5, Figure 6, Figure 7, Figure 8, Figure 9, Figure 10 and Figure 11 show the spatial distribution of ionospheric TEC on DOY080, DOY172, DOY266, and DOY356 in 2014 and 2019. As the earth rotates and revolves in an elliptical orbit around the sun, the ionospheric TEC at mid latitude and low-latitude has obvious diurnal and seasonal variations. Within one day, the ionospheric TEC generally peaks at noon (12:00–14:00 local time) and reaches its trough at night or morning. It can be clearly seen that the TEC peak area continues to move westwards. The diurnal variation of TEC in Japan is greatly affected by solar activities. The TEC peak (3:00–5:00 UTC) in the high solar activity year can reach 2–3 times that of the low solar activity year, while the TEC in most areas at night drops below 5 TECu.

At the same time, the TEC in Japan increase as the latitude decrease, and the range of variations is large. In the daytime of the high solar activity year (2014), there was higher variation range of TEC between mid-latitude and low latitude, and the contour also became dense. In the low solar activity year (2019), the TEC varied slightly with latitude. In addition, the variation of TEC with latitude was greater during the day than at night.

Comparing Figure 5 with Figure 7, we can find the peak of TEC on summer solstice (about 40 TECu) is lower than winter solstice (about 80 TECu), which has indicated obvious winter anomaly in 2014. Winter anomaly generally only appears during the day. This is due to the solar radiation, the thermal effect in thermosphere resulted in an increase in the amount of ionization and diffusion of neutral component, which is blown to the other hemisphere, and the difference in the O/N_2_ concentration ratio in summer and winter of high solar activity year increased.

### 4.2. Temporal Variation Characteristics of TEC in Japan Regions

**Figure 12:**
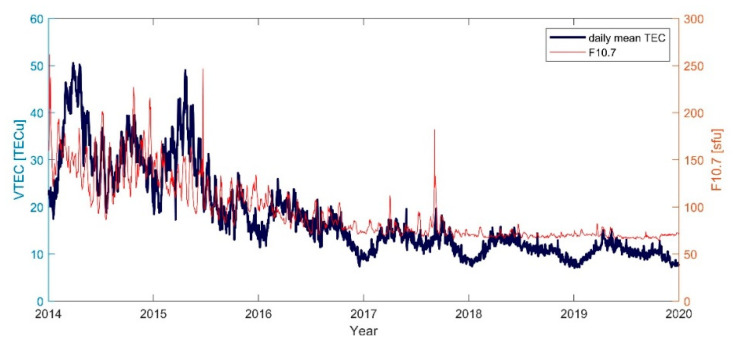
Time series of daily mean TEC and solar activity index.

Figure 12 illustrates the daily average TEC in Japan from 2014 to 2019 calculated by the SCH model. The correlation coefficient between it and the F10.7 index is calculated to be 0.89. It can be seen that there is a strong correlation between the ionospheric TEC and the solar activities from high solar activity years to low solar activity years. The F10.7 reached its highest point in 2014, while the ionospheric TEC in Japan also reached its peak in 2014. After that, as solar activity decreased, TEC also tended to decrease year by year. There were two peaks of TEC in Japan every year in March–April and September–October, showing a semiannual periodicity. TEC reached the first trough in about January, then gradually rose and reached the first peak in March–April; after that TEC decreased to the second trough in June–July, and then rose to the second peak in September–October, finally decreased until December–January. The peak and trough times were roughly the same as the equinoxes and solstices. The equinoxes’ peaks of TEC are probably related to Russell-McPherron effect [32], manifesting that twice as many storms occur on average during the equinoctial months as during the solstitial months. The energy deposited at the solstices can increase by 40% in the average energy input during a storm at the equinoxes. The semiannual periodicity of TEC variation causing by geomagnetic activities can be split into two annual variations, one peaking in spring and one in fall.

**Figure 13:**
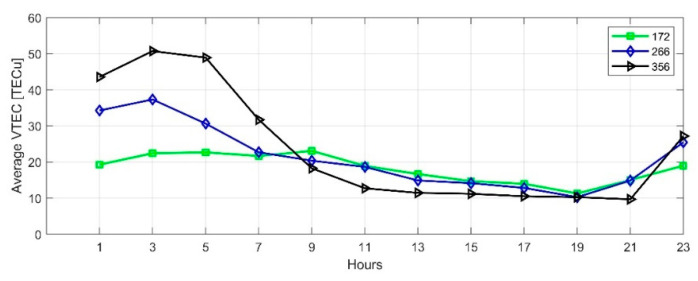
Time series of TEC in Japan with 2-h resolution on summer solstice day, autumnal equinox day, winter solstice day of 2014.

**Figure 14:**
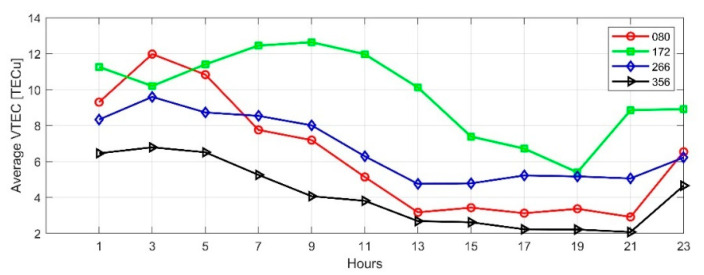
Time series of TEC in Japan with 2-h resolution on vernal equinox day, summer solstice day, autumnal equinox day, winter solstice day of 2019.

We used the SCH model to calculate TEC of the selected date in Section 4.1. Figure 13 and Figure 14 show the diurnal variations in equinoxes and solstices of the high solar activity year (2014) and low solar activity year (2019), respectively. Affected by solar activities, the ionospheric TEC in 2014 was significantly higher than in 2019. When the ionospheric TEC is in a calm state, it is the smallest in winter and the largest in summer, when the sun has the highest angle over horizon in Japan. On DOY356 in 2014, the disturbance storm time (Dst) index decreased to −51nT, and an ionospheric storm occurred, which increased the diurnal variation of TEC on that day.

## 5. Analysis of Ionospheric Disturbances Prior to 2016 Kumamoto Earthquake

The Mw 7.0 earthquake occurred in Kumamoto, Japan, at 16:25:06 UT on 15 April. This is the highest-level earthquake in the history of local observation in Kyushu, Japan. The VTEC several days before and after the earthquake was calculated using GNSS dual-frequency observation data from multiple IGS permanent stations near the epicenter, and the median *m* and standard deviation *σ* were calculated with *m* ± 1.5*σ* as the boundary. If the VTEC is higher or lower than the boundary, there may be an ionospheric disturbance. It is necessary to comprehensively analyze the ionospheric disturbance conditions prior to the earthquake after eliminating the factors of solar and geomagnetic activities.

**Figure 15:**
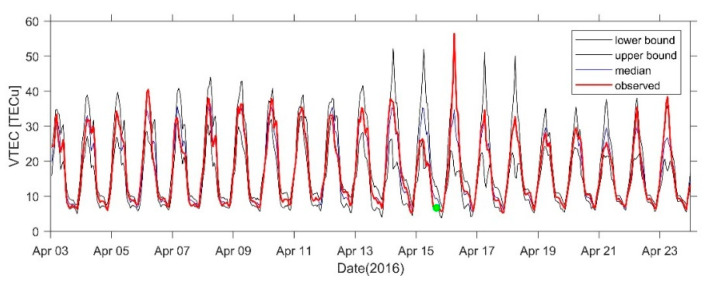
VTEC time series from 3 to 23 April at GMSD Station. The green dots indicate the time of the earthquake.

**Figure 16:**
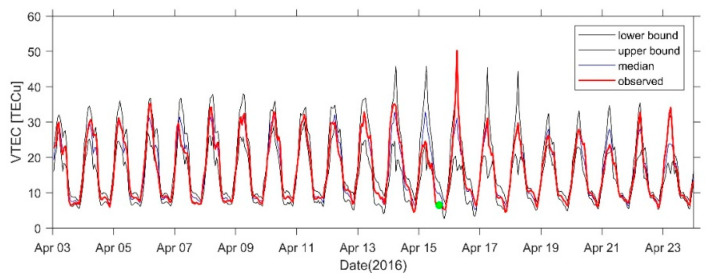
VTEC time series from 3 to 23 April at AIRA Station. The green dots indicate the time of the earthquake.

**Figure 17:**
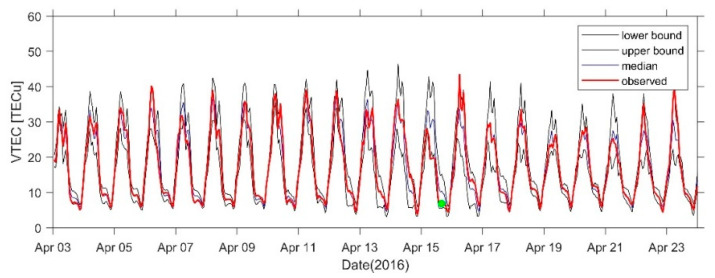
VTEC time series from 3 to 23 April at SHAO Station. The green dots indicate the time of the earthquake.

**Figure 18:**
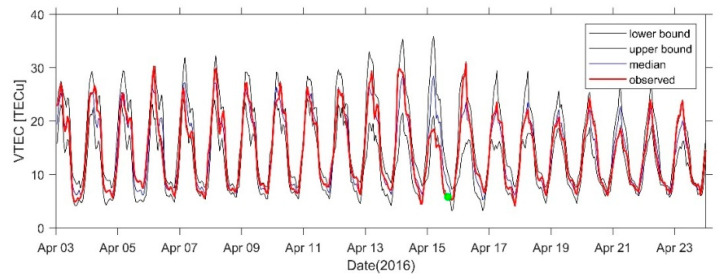
VTEC time series from 3 to 23 April at TSK2 Station. The green dots indicate the time of the earthquake.

Figure 15 shows the 21-day VTEC time series before and after the earthquake at the GMSD station closest to the epicenter. Positive disturbances occurred in the TEC at some times on 4 April, 6 April, 10 April, and 14 April, and the range was 3–5 TECu. An obvious positive disturbance occurred on 16 April. A negative disturbance of −5 TECu occurred on 7 April and a significant negative disturbance of −10 TECu occurred on 15 April. Figure 16, Figure 17 and Figure 18 show the time series at AIRA, SHAO, and TSK2 stations which are slightly far from the epicenter. We can see a similar phenomenon to Figure 15. In addition, the VTEC was also higher or lower on some days after the earthquake.

**Figure 19:**
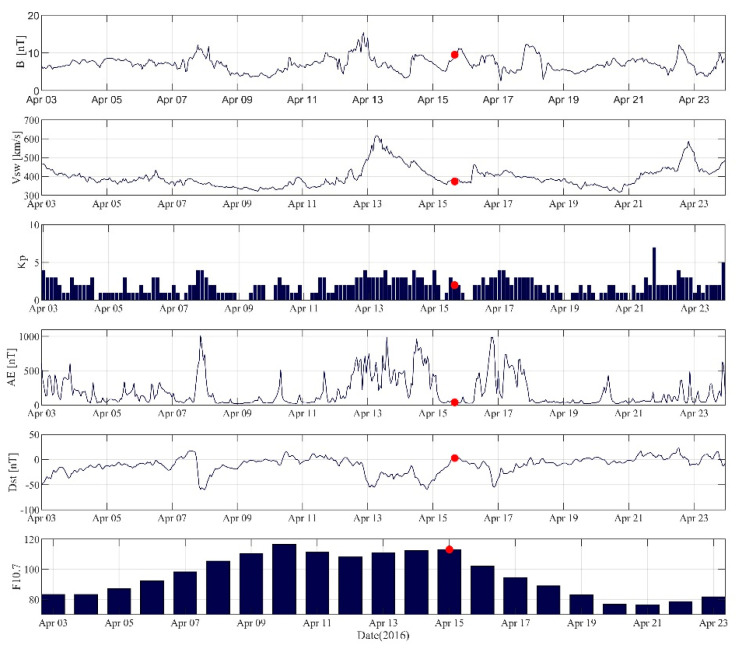
Solar and geomagnetic indices from 3 to 23 April 2016. The red dots indicate the time of the earthquake.

Figure 19 shows the interplanetary magnetic field (IMF) index, solar wind speed (VSW), geomagnetic K-indices (KP), auroral electrojet (AE) index, disturbance storm time (Dst) index, solar radio flux at 10.7cm (F10.7) from 3 to 23 April. The red dots indicate the time of the earthquake. As seen from the figure, F10.7 gradually increased and it was above 100 sfu from 10 to 15 April, and it began to decrease from 16 April. Therefore, the ionospheric TEC disturbances within 5 days before the earthquake were required to be specially analyzed. VSW was lower than 400 km/s most of the time. It rose sharply on 13 April, reaching 600 km/s, and then dropped below 400 km/s on 15 April. The IMF shocked on 7–8 and 13 April, and then rose several times. The K_P_ index reached 4 on 7 April and 13–14 April. Dst dropped rapidly below −50 nT on 3, 7–8, and 13–14 April, and the AE index also rose sharply, indicating that medium intensity geomagnetic storm was likely to occur these days, whereas the magnetic field was relatively calm on the remaining days.

The reasons for the ionospheric TEC disturbances were analyzed according to the solar and geomagnetic indices before the earthquake. From 7 to 8 April, Dst index dropped rapidly to below −50 nT, KP and AE rose sharply, and |B| also rose. On these days, the TEC had a disturbance of −5 TECu, indicating that a geomagnetic storm occurred. F10.7 reached its peak on 10 April, approaching 120 sfu, and AE also rose slightly. It may be that the solar activity caused the positive ionospheric TEC disturbance. AE had volatile fluctuation from 13 to 14 April, accompanied by a decrease in Dst and a sharp increase in V_SW_, indicating that the solar activity might have caused the disturbance of the geomagnetic field. On 15 April, the recovery phase of the geomagnetic storm, V_SW_ dropped to below 400 km/s, AE was less than 100 nT, Dst was rising towards 0, and K_P_ was less than 3. It can be concluded that the geomagnetic field was not so active on this day. On the same day, a negative disturbance of −10 TECu occurred, which was significantly greater than the other days, and the VTEC variations of each station were very similar. According to the above analysis, it can be found that the TEC did have disturbances before the Kumamoto earthquake. The disturbance was the largest on 15 April, and there was no geomagnetic storm that day. However, F10.7 on that day reached 113.2, and the solar radiation was relatively strong. Further analysis is needed to determine whether the ionospheric TEC disturbance on that day was related to solar activity.

**Figure 20:**
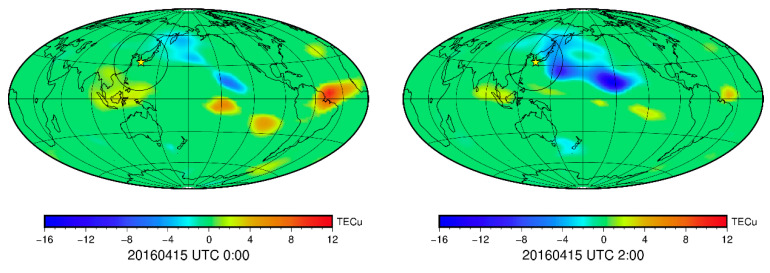

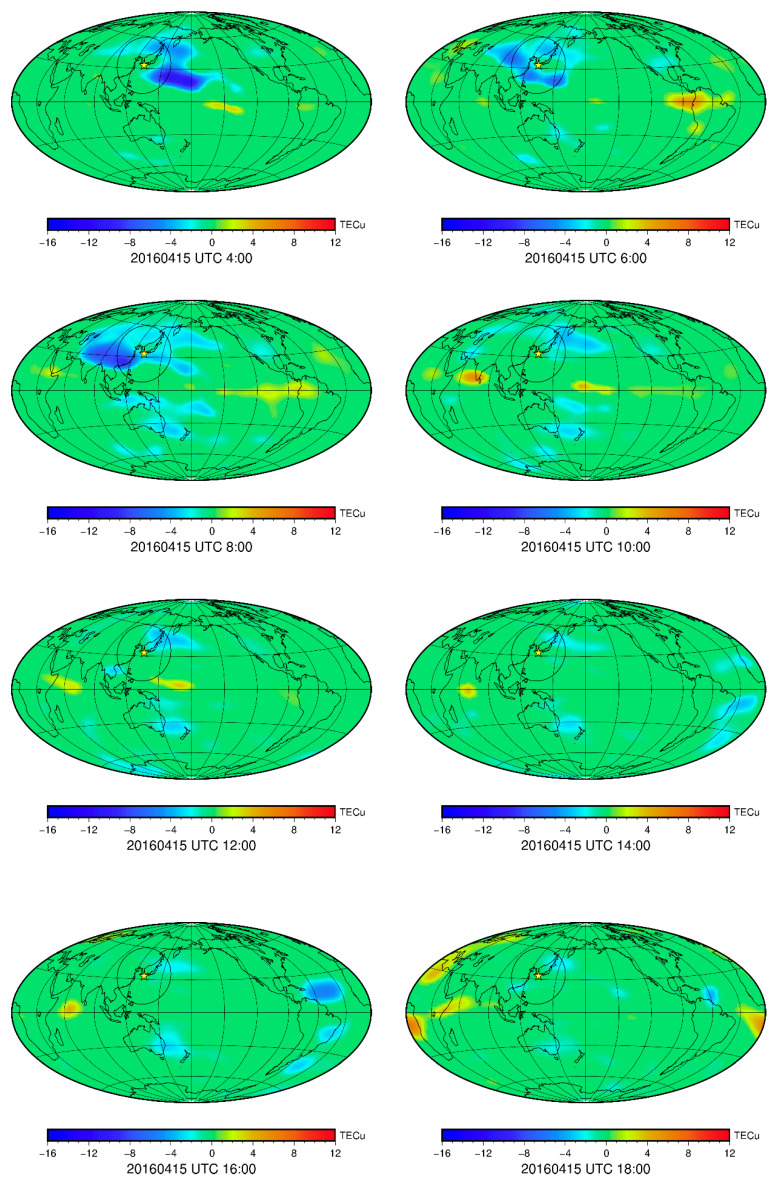
Global anomalies of TEC at 2-h intervals on 15 April 2016.

In order to further analyze whether the TEC disturbance on 15 April is related to the earthquake, we used the VTEC within 10 days before the earthquake as the background, and calculated the median *m* and standard deviation *σ*. If the VTEC on 15 April is higher or lower than *m* ± 2*σ* at the corresponding time, there may be the TEC disturbance. The global TEC disturbances at the 2-h interval between 0:00 UTC and 18:00 UTC on April 15 are shown in Figure 20. The yellow ★ is the location of the epicenter, and the black ellipse is the influence range of the Kumamoto earthquake calculated according to the Dobrovolsky formula [33]. At 0:00 UTC, there is no obvious TEC disturbance in Japan. At 2:00 UTC, a negative TEC disturbance of −3–−4 TECu appeared in northeast Japan, and another negative disturbance of −4–−8 TECu appeared in southeast Japan. The disturbance reached its maximum at 25 °N and 155 °E, then moved westwards slowly in the influence range of the earthquake. At 4:00 UTC, the negative TEC disturbance covered northern and southeastern Japan. At 6:00 UTC, the negative TEC disturbance area in northern and southern Japan expanded significantly, gradually covering most of Japan and approaching the epicenter. After that, the TEC disturbance remained at −2–−4 TECu and gradually weakened from 14:00 UTC to 18:00 UTC when the TEC disturbance in the area affected by the Kumamoto earthquake completely disappeared.

The TEC disturbance lasted for about 12 h, and it was negative and distributed near the epicenter. There was no large-scale negative TEC disturbance in other areas of the world. The TEC disturbances caused by solar activities are often global and positive, so it was not related to solar activity and solar-terrestrial environment this time, but may be one of the precursors of the earthquake. In addition, the TEC disturbance area did not cover the epicenter, but was mainly in the north and south of the epicenter, where the side closer to the equator was larger. The ionospheric disturbance shape this time is consistent with other research results [34,35]. There are positive disturbances before most earthquakes, but the negative disturbance appeared in the seismogenic zone before the Kumamoto earthquake. The main reason may be the rupture of rock holes in the crust of the seismogenic zone before the earthquake. During the process of radon overflowing from the surface and spreading to the atmosphere, it was affected by various factors such as solar radiations, temperature, humidity, and light, generating abnormal electric field at lower end of ionosphere and resulting in different ionospheric disturbances [14].

## 6. Observing Ionization Blobs in Japan Region

The ionosphere is composed of plasma ionized gas containing positive ions and free electrons. During a geomagnetic storm, it will produce irregular disturbances, and any disturbance of plasma density will cause interference to GNSS and radar signals. These disturbances or irregularities are manifested as a partial loss (bubbles) or partial increases (blobs) of electrons [17]. These phenomena not only appear in high latitudes, but also in middle and low latitudes. This part of the ionosphere occupies a small part of the atmosphere and coexists with the thermosphere, about 80–400 km from the earth’s surface.

**Figure 21:**
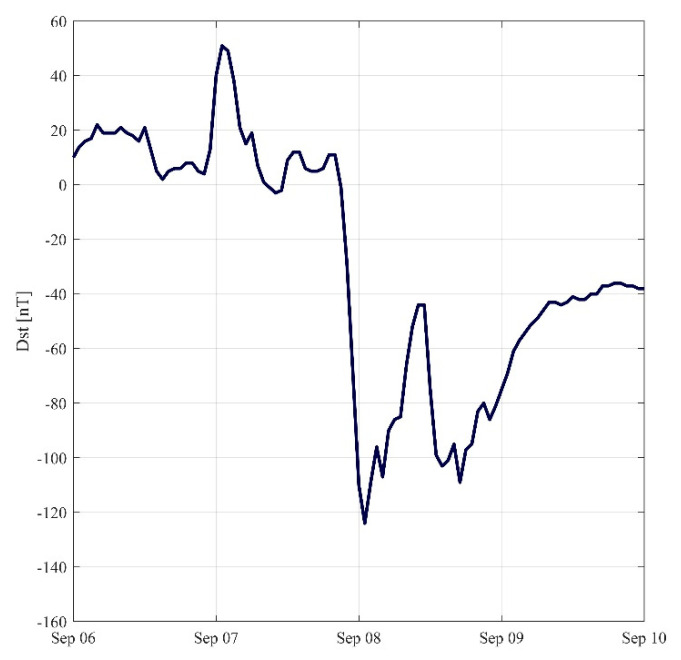
Time series of Dst index from 6 to 9 September 2017.

In this section, we extracted the Dst index time series from 6 to 9 September 2017. As shown in Figure 21, Dst dropped sharply to −124 nT at 2:00 UTC on 8 September, and dropped to −109 nT again at 18:00 UTC, indicating that violent geomagnetic activity occurred from 7 to 8 September, possibly accompanied by a geomagnetic storm. In order to detect the possible ionized blobs, bubbles and small-scale scintillation during geomagnetic storms in Japan, the TEC variations on 8 September were inverted to observe this type of phenomenon.

**Figure 22:**
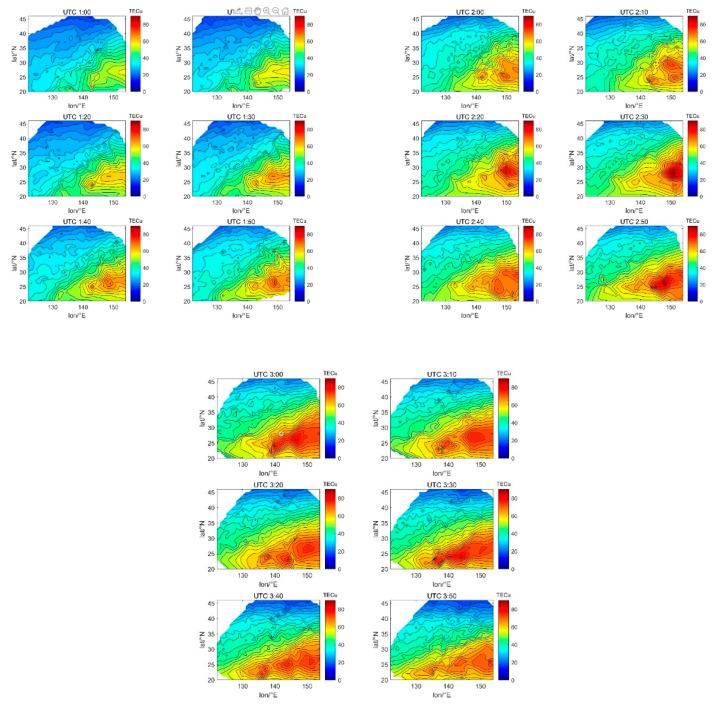
TEC variations with 10-min resolution from UTC 1:00 to UTC 3:50 on 8 September 2017.

According to the TEC inversion results, the TEC variations in Japan with a resolution of 10 min on 8 September are shown in Figure 22. The small-scale blobs were observed at approximately 1:40 UTC, with a peak of 60–70 TECu. From 2:10 to 2:50 UTC, several blobs of varying sizes were forming, one of which was growing and moving westward, with a peak of 80–90 TECu. From 3:00 UTC to 3:40 UTC, the ionization blobs gradually split into three, and then they became smaller and disappeared. Most of the blobs occurred in low latitudes, probably because blobs are more likely to form in low latitudes and high latitudes than bubbles, which has been proven by previous research [36]. In addition, there are obviously more small-scale blobs than large-scale blobs, which is consistent with the research results in [37]. Figure 22 clearly shows the complete evolution of ionized blobs in Japan, indicating that the GNSS ionospheric model can effectively monitor such space weather phenomena.

## 7. Conclusions

In this paper, we studied the modeling of the ionosphere in Japan and its spatiotemporal distribution characteristics, as well as the ionospheric disturbances before the earthquake and ionization blobs during the geomagnetic storm. It aims to comprehensively study the ionospheric variations in Japan and provide guidance for the study of other regions. We started to use GEONET data to calculate the ionospheric TEC in Japan, using more than 1200 GEONET receivers for parameter fitting and model evaluation, selecting 100 receivers as verification stations, and compared performance of different regional models in Japan. It was found that the SCH model performs better in Japan, with an average bias within 1.0 TECu and an RMS error within 3.5 TECu.

The ionospheric TEC in Japan shows different diurnal variations under different solar activity conditions. It increases as latitude decreases and is also related to solar activity. There are two peaks and troughs of TEC during a year in Japan, with the highest in spring and summer and the lowest in winter. TEC has a strong correlation with solar activity, reaching its maximum in 2014 and minimum in 2019.

The analysis of the 21-day VTEC time series calculated by data from multiple stations near the epicenter before and after the earthquake combined with the space weather environment confirmed that the negative ionospheric disturbances on 15 April was related to the Kumamoto earthquake, and the shape and reason of the ionospheric disturbance was explored. It is shown that the ionospheric disturbance before the earthquake can be used as a basis for earthquake forecasting. In addition, we traced the generation and evolution of ionization blobs in Japan during geomagnetic storms, which provided an example for monitoring space weather phenomena by the ionospheric disturbance.

## Data Availability

The GEONET observation data used in this paper can be accessed at ftp://terras.gsi.go.jp (accessed on 15 March 2021) after application. The IGS observation data can be accessed at ftp://gdc.cddis.eosdis.nasa.gov (accessed on 15 March 2021). The links for these data can be accessed all the time.
